# Biologic Therapies for Knee Osteoarthritis: A Structured Narrative Review of Joint Preservation Strategies and Clinical Outcomes

**DOI:** 10.7759/cureus.114564

**Published:** 2026-08-15

**Authors:** Santiago Aguayo-Pigeonutt, Alan Mendoza-Castañeda, Iker Fernández-Ugalde, Kepler Garcia-Noriega, Santiago Flores-Cabrera, María-Fernanda Gallegos-Sánchez, Miranda Freijo-Valdovinos, Airam A Arias Villaverde, Rodrigo Anaya-Eulogio, José Emiliano González Flores

**Affiliations:** 1 School of Medicine and Health Sciences, Tecnológico de Monterrey, Mexico City, MEX; 2 Department of Orthopedics, Centro Médico ABC, Mexico City, MEX; 3 Medical Research and Academic Mentorship, ShareTheMed Research Group, Mexico City, MEX; 4 Faculty of Public Health, Universidad Anáhuac México, Mexico City, MEX

**Keywords:** bone marrow aspirate concentrate, intra-articular therapy, joint preservation, knee osteoarthritis, mesenchymal stromal cells, narrative review, orthobiologics, personalized medicine, platelet-rich plasma, regenerative medicine

## Abstract

Knee osteoarthritis (KOA) is among the leading causes of chronic pain and physical disability worldwide, representing a major clinical challenge as currently available treatments primarily address symptoms rather than the biological mechanisms driving disease progression. As interest in joint preservation strategies has grown, biologic therapies have emerged as promising approaches capable of modulating the intra-articular environment and potentially delaying invasive interventions. This structured narrative review critically synthesizes contemporary evidence retrieved from PubMed, the Cochrane Library, and Google Scholar to evaluate the biological rationale, clinical applications, therapeutic outcomes, and current limitations of biologic therapies for KOA. Collectively, the available literature suggests that platelet-rich plasma, mesenchymal stromal cell-based therapies, and bone marrow aspirate concentrate are associated with meaningful improvements in pain, physical function, and patient-reported outcomes, particularly in appropriately selected patients and when incorporated into individualized treatment strategies. However, substantial heterogeneity in product composition, preparation techniques, and patient selection continues to limit reproducibility and hamper standardized clinical recommendations. Although the available evidence supports symptomatic improvements with selected biologic therapies, their long-term structural effects and disease-modifying potential remain uncertain. Current evidence therefore supports their consideration as adjunctive options within individualized joint-preservation strategies rather than as replacements for established surgical treatment. Further high-quality comparative studies and greater standardization of treatment protocols and patient selection are needed to define their long-term clinical role.

## Introduction and background

Knee osteoarthritis (KOA) is a leading cause of chronic pain, functional limitation, and disability worldwide, with a substantial clinical and socioeconomic burden, particularly among aging populations [1,2]. Rather than a simple “wear-and-tear” disorder, KOA is now recognized as a complex whole-joint disease involving articular cartilage degeneration, subchondral bone remodeling, synovial inflammation, meniscal and ligamentous dysfunction, and dysregulation of the intra-articular environment [2]. This broader pathophysiological understanding has supported growing interest in biologic and orthobiologic therapies aimed at modulating the joint environment and preserving native joint function.

Despite increasing clinical use, the role of biologic therapies in KOA remains incompletely defined. Considerable heterogeneity exists in product composition, preparation techniques, cellular source, platelet and leukocyte concentration, dose, administration schedules, patient selection, comparators, and follow-up duration [1-7]. Platelet-rich plasma (PRP) protocols vary substantially in platelet concentration, leukocyte content, activation methods, and dosing schedules [1,3,4], while mesenchymal stromal cell (MSC)-based therapies differ according to tissue source, processing methods, cellular dose, and concomitant interventions [2,5]. Bone marrow aspirate concentrate (BMAC) preparations are similarly affected by differences in aspiration technique, processing systems, cellular yield, and biological composition [6,7]. This variability limits reproducibility and complicates direct comparison across studies.

Although PRP, MSC-based therapies, and BMAC have demonstrated improvements in pain and function, the magnitude and durability of benefit remain variable [1-7]. Evidence supporting structural preservation, cartilage regeneration, or definitive disease modification is considerably less certain, and limited head-to-head comparisons prevent the establishment of a reliable therapeutic hierarchy [1,2,6,7]. Accordingly, an integrated appraisal of clinical effectiveness, biological rationale, patient selection, and methodological limitations is needed to define the contemporary role of these therapies within joint-preservation strategies.

This structured narrative review synthesizes the current evidence on biologic therapies for KOA, with particular emphasis on biological rationale, clinical effectiveness, safety, patient-selection factors, methodological limitations, and emerging therapeutic directions. By integrating evidence on PRP, MSC-based therapies, BMAC, and related orthobiologic approaches, this review aims to provide a clinically relevant framework for evidence-informed and individualized joint-preservation decision-making.

## Review

Methods

Study Design

This study was conducted as a structured narrative review aimed at critically synthesizing contemporary clinical evidence on biologic and orthobiologic therapies for KOA. A predefined methodological framework was used to promote transparency and consistency across literature identification, study selection, data extraction, methodological appraisal, and narrative synthesis. Because this review was not designed as a systematic review or meta-analysis, the literature search and study-selection process were intended to identify representative and clinically relevant evidence rather than to achieve exhaustive retrieval of all available publications or formal PRISMA compliance. The literature identification and selection process was summarized in a descriptive flow diagram to improve transparency. Given the anticipated clinical and methodological heterogeneity across biologic products, preparation techniques, dosing regimens, patient populations, comparator interventions, follow-up periods, and outcome measures, quantitative pooling was not planned, and the evidence was synthesized narratively.

Information Sources and Search Strategy

A structured literature search was conducted in PubMed, the Cochrane Library, and Google Scholar to identify studies evaluating biologic and orthobiologic therapies for KOA. The search encompassed articles published between January 1, 2015, and March 1, 2026. PubMed and the Cochrane Library served as the principal biomedical information sources, whereas Google Scholar was used as a complementary platform to identify potentially relevant studies not retrieved through the primary database searches.

The search strategy combined controlled vocabulary and free-text terms related to KOA and biologic or orthobiologic interventions. Database-specific search approaches, information sources, publication limits, and complementary identification methods are summarized in Table 1.

**Table 1:** Literature search strategy and information sources.

Information source	Search strategy	Filters applied
PubMed	("Osteoarthritis, Knee"[Mesh]) AND ("Platelet-Rich Plasma"[Mesh] OR "Mesenchymal Stem Cells"[Mesh]) AND (Randomized Controlled Trial[pt] OR "systematic review" OR "meta-analysis")	Published within the last 10 years; free full text; English; humans; adults aged ≥19 years
The Cochrane Library	("knee osteoarthritis" OR "osteoarthritis of the knee") AND ("platelet rich plasma" OR PRP OR "mesenchymal stem cells" OR MSC OR "bone marrow aspirate concentrate" OR BMAC OR "adipose derived stem cells" OR orthobiologics) AND (WOMAC OR KOOS OR "clinical outcomes" OR "joint preservation")	No additional filters applied
Google Scholar	"knee osteoarthritis" ("platelet rich plasma" OR PRP OR "mesenchymal stem cells" OR MSC OR "bone marrow aspirate concentrate" OR BMAC OR "adipose derived stem cells" OR orthobiologics) (WOMAC OR KOOS OR "clinical outcomes" OR "joint preservation")	Publication years: 2016-2025

Eligibility Criteria

Eligible publications included both primary clinical investigations and evidence syntheses because they served complementary purposes within this structured narrative review. Primary studies were used to characterize individual interventions, patient populations, treatment protocols, clinical outcomes, structural findings, and safety events. Systematic reviews and meta-analyses were incorporated to evaluate the consistency, direction, and methodological limitations of the broader evidence base and to contextualize findings from individual clinical studies. Evidence syntheses were not treated as additional independent patient cohorts, and their reported sample sizes were not combined with those of the primary studies. The predefined inclusion and exclusion criteria applied during study selection are summarized in Table 2.

**Table 2:** Inclusion and exclusion criteria. KOA, knee osteoarthritis

Eligibility category	Criterion
Inclusion	Primary clinical studies (randomized controlled trials, comparative studies, cohort studies, case-control studies, and case series)
Inclusion	Systematic reviews and meta-analyses
Inclusion	Studies evaluating biologic or orthobiologic therapies for KOA
Inclusion	Studies reporting at least one predefined clinical or safety outcome
Inclusion	Full-text articles published in English or Spanish
Exclusion	Animal, in vitro, or laboratory-based studies
Exclusion	Non-KOA studies without separate knee-specific data
Exclusion	Studies evaluating nonbiologic interventions
Exclusion	Editorials, letters, conference abstracts, and expert opinions
Exclusion	Duplicate reports or studies with insufficient methodological or outcome data

Interventions of interest included PRP, MSC-based therapies, BMAC, microfragmented adipose tissue (MFAT), stromal vascular fraction (SVF), adipose-derived stromal products, combined biologic approaches, and other intra-articular regenerative therapies used in the management of KOA. When multiple reports described the same cohort, the most complete or most recent publication was retained.

Study Selection

Study selection was guided by clinical relevance, methodological quality, and contribution to the principal themes of the review. Higher-level evidence, including systematic reviews, meta-analyses, and randomized controlled trials, was prioritized when available, whereas observational and early-phase studies were included to contextualize emerging therapies, patient-selection factors, and preliminary structural findings. The included publications were intended to provide a representative and clinically informative evidence base rather than an exhaustive collection of all available studies.

A structured selection framework was used to promote transparency and consistency during literature identification, screening, eligibility assessment, data extraction, and narrative synthesis. Because the study was designed as a structured narrative review rather than a formal systematic review or meta-analysis, the selection process was not intended to achieve exhaustive retrieval or formal PRISMA compliance. Instead, representative clinical evidence was integrated across heterogeneous therapeutic products, study designs, patient populations, comparators, follow-up periods, and outcome measures.

**Figure 1:**
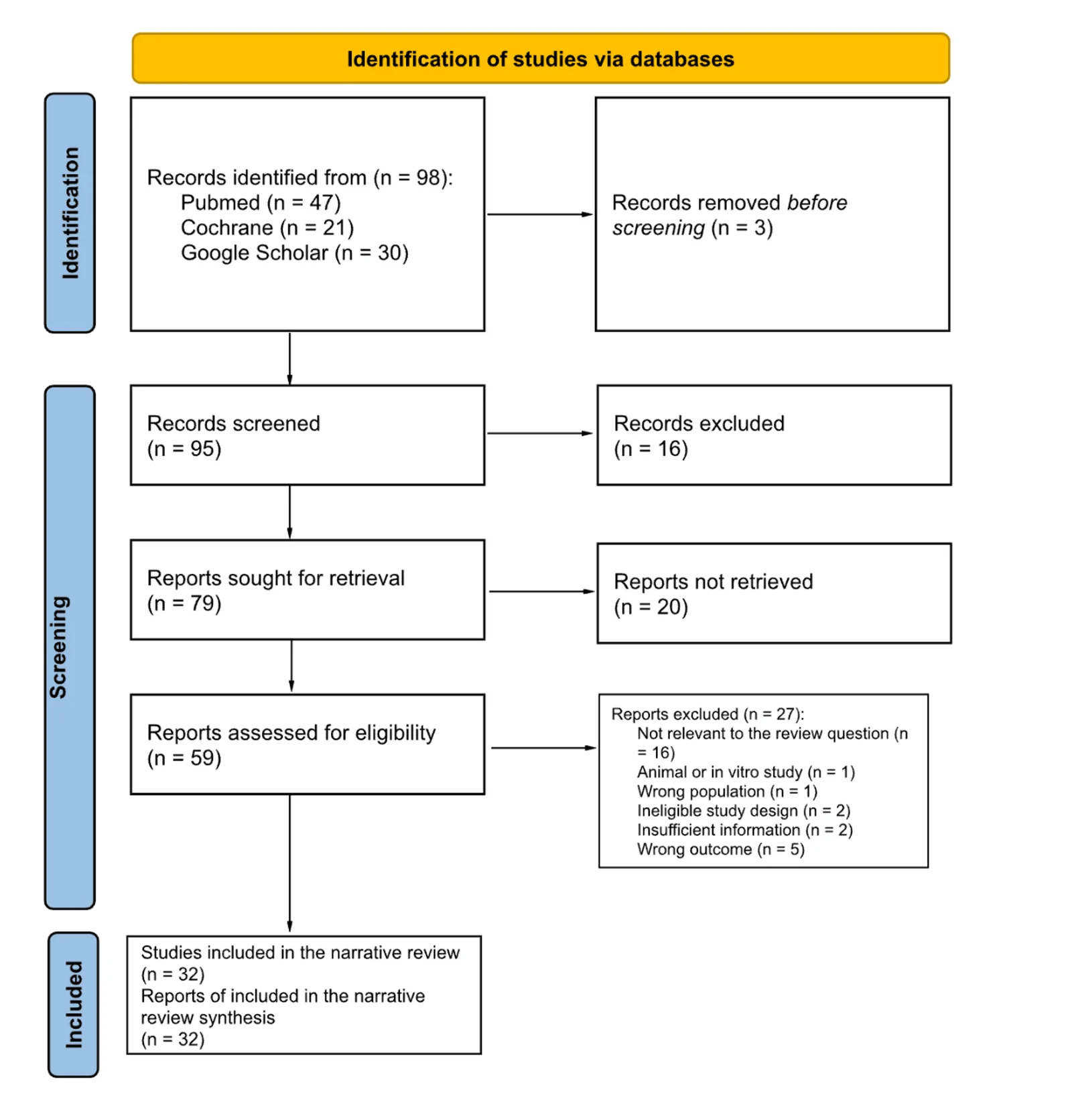
Literature identification and selection flow diagram. Flow diagram illustrating the structured process of literature identification, screening, full-text assessment, and selection for the narrative synthesis. Reasons for full-text exclusion are presented to enhance methodological transparency. Reports classified as not retrieved corresponded to records for which complete full-text access could not be obtained through the available institutional and electronic sources.

The literature identification and selection process is summarized descriptively in Figure 1 to illustrate how the literature incorporated into the narrative synthesis was identified and selected; the diagram is not intended to represent formal PRISMA reporting or an exhaustive systematic search. The review was not prospectively registered, and quantitative pooling was not planned because of the substantial clinical and methodological heterogeneity across interventions, populations, study designs, and reported outcomes.

Data Extraction

Data were extracted using a standardized collection form developed before full-text review to ensure consistency across included publications. Extracted variables included study design, publication year, country, sample size, patient demographics, osteoarthritis severity, Kellgren-Lawrence (KL) classification, intervention type, biologic source, preparation and processing methods, reported concentration or cellular dose, number and frequency of administrations, comparator intervention, follow-up duration, and reported clinical, structural, and safety outcomes.

Pain and functional outcomes were documented using the instruments reported by the original studies, including the Visual Analog Scale (VAS), the Western Ontario and McMaster Universities Osteoarthritis Index (WOMAC) [8], and the Knee Injury and Osteoarthritis Outcome Score (KOOS) [9]. Additional data included quality-of-life measures, imaging findings, structural progression, biomarker outcomes, conversion to arthroplasty, and treatment-related adverse events. Data extraction was conducted independently from the interpretive synthesis to preserve methodological consistency and avoid premature comparative inference.

Methodological Appraisal

Because this study was designed as a structured narrative review, formal study-level risk-of-bias instruments and certainty-of-evidence frameworks were not applied. Instead, methodological characteristics such as study design, sample size, comparator selection, follow-up duration, intervention heterogeneity, and completeness of outcome reporting were considered qualitatively when interpreting the evidence. Higher-level comparative evidence was given greater interpretive weight, whereas observational and early-phase studies were used primarily to contextualize emerging therapies and preliminary findings.

These variables were considered when evaluating the reliability, generalizability, and clinical relevance of the reported findings. Particular caution was applied when interpreting evidence derived from small cohorts, nonrandomized designs, short follow-up periods, heterogeneous intervention protocols, or incompletely reported outcomes.

This appraisal was intended to support critical narrative interpretation rather than to generate formal study-level risk-of-bias classifications or certainty-of-evidence ratings. Accordingly, methodological characteristics were considered qualitatively, and no study was excluded solely on the basis of an aggregate quality score.

Data Synthesis

Primary clinical studies constituted the principal source for intervention-specific outcomes, including pain, physical function, imaging findings, durability of response, and adverse events. Systematic reviews and meta-analyses were used to assess whether these findings were consistent across the broader literature and to identify areas of agreement, uncertainty, and methodological heterogeneity. To reduce the risk of double counting, sample sizes reported in evidence syntheses were not aggregated with those of primary studies, and conclusions were based on the convergence and quality of findings rather than on the cumulative number of participants represented across publications.

The synthesis prioritized recurring clinical patterns, consistency of findings across studies, and the relationship between biological mechanisms and patient-level outcomes. Particular emphasis was placed on distinguishing reproducible symptomatic benefits from preliminary structural signals and on interpreting treatment effects within the context of disease severity, patient phenotype, and intervention heterogeneity. The final synthesis therefore focused on defining the contemporary role of orthobiologic therapies within personalized, multimodal joint-preservation strategies rather than establishing a definitive hierarchy among interventions.

Results

The narrative synthesis incorporated 32 clinically relevant publications selected to represent the contemporary evidence across the principal orthobiologic therapies evaluated orthobiologic therapies for KOA. The evidence base included systematic reviews, meta-analyses, randomized controlled trials, prospective comparative studies, cohort investigations, and other clinically informative original studies evaluating PRP, MSC-based therapies, BMAC, MFAT, SVF, and other emerging regenerative approaches. Primary clinical investigations were used to characterize intervention-specific outcomes, treatment protocols, patient-selection factors, structural findings, and safety events, whereas systematic reviews and meta-analyses were used to contextualize the consistency and certainty of the broader evidence. These two levels of evidence were therefore interpreted as complementary rather than cumulative, and no attempt was made to aggregate their reported sample sizes. Together, these publications provided the basis for the following narrative synthesis of the biological rationale, clinical effectiveness, patient selection, safety, limitations, and future directions of orthobiologic interventions in KOA [1-3,5,7,10-36]. Additional references cited elsewhere in the manuscript were used exclusively to support contextual, mechanistic, or methodological statements and were not considered part of the formal evidence set. The principal methodological and clinical characteristics of the included publications are summarized in Table 3.

**Table 3:** General characteristics and evidentiary roles of representative publications included in the narrative synthesis. Primary clinical investigations were used to characterize intervention-specific clinical, structural, and safety outcomes. Systematic reviews and meta-analyses were included to contextualize the consistency, direction, and methodological certainty of the broader evidence base. Evidence syntheses were not considered independent patient cohorts, and their sample sizes were not aggregated with those reported in primary studies. AD-MSC, adipose-derived mesenchymal stromal cell; BMAC, bone marrow aspirate concentrate; BM-MSC, bone marrow-derived mesenchymal stromal cell; HA, hyaluronic acid; IKDC, International Knee Documentation Committee; KOA, knee osteoarthritis; KOOS, Knee Injury and Osteoarthritis Outcome Score; MFAT, microfragmented adipose tissue; MSC, mesenchymal stromal cell; PRP, platelet-rich plasma; ROM, range of motion; UC-MSC, umbilical cord-derived mesenchymal stromal cell; VAS, Visual Analog Scale; WOMAC, Western Ontario and McMaster Universities Osteoarthritis Index

Study	Study design	Biologic intervention	Comparator	Evidence base/sample	Follow-up	Main outcomes	Key clinical interpretation
Auroux et al. [1]	Systematic review and meta-analysis of placebo-controlled trials	Intra-articular PRP	Saline placebo	1,616 participants	3-12 months	VAS and WOMAC	PRP was associated with short- to intermediate-term symptomatic improvement, although clinically relevant benefit was less consistent at 12 months.
Chen et al. [2]	Systematic review and network meta-analysis	BM-MSCs, AD-MSCs, and UC-MSCs	HA, placebo, or conventional treatment	585 participants	6-48 months	Pain, WOMAC, ROM, and imaging outcomes	Clinical and structural responses differed according to cellular source, but heterogeneity prevented establishment of a definitive hierarchy.
Zhuang et al. [3]	Randomized comparative clinical trial	One, three, or five PRP injections	Alternative PRP dosing schedules	120 participants; 106 completed follow-up	52 weeks	VAS and WOMAC	Multiple-injection protocols produced more sustained improvement than a single injection, while five injections did not clearly outperform three.
Song et al. [5]	Systematic review and meta-analysis	MSC-based therapies from different tissue sources	Saline, HA, PRP, or standard treatment	584 participants	Predominantly 6-12 months	Pain, function, and safety	MSC-based therapies were associated with symptomatic and functional improvement, although substantial heterogeneity reduced certainty.
Dulic et al. [7]	Prospective comparative clinical study	BMAC	PRP and HA	150 participants	12 months	VAS and WOMAC	BMAC, PRP, and HA produced clinical improvement, with findings supporting the potential value of biologically complex preparations while not establishing universal superiority.
Hong et al. [10]	Systematic review and meta-analysis	Intra-articular PRP	HA, placebo, or other controls	Multiple clinical studies	Predominantly ≤12 months	Pain, function, and adverse events	PRP demonstrated an overall favorable clinical and safety profile, with responses varying according to preparation and comparator.
Di Matteo et al. [11]	Systematic review of clinical evidence	Minimally manipulated cellular products, including BMAC and adipose-derived preparations	Variable comparators	Multiple clinical studies	Variable	Pain, function, structural outcomes, and safety	Minimally manipulated cellular therapies showed clinical potential, but product heterogeneity and limited standardization restricted comparisons.
Kyriakidis et al. [12]	Systematic review	MSC-based therapies for early-to-moderate KOA	Conservative or active controls	539 participants	3-60 months	VAS, WOMAC, KOOS, and IKDC	More favorable responses were observed in early-to-moderate disease, emphasizing the relevance of residual joint reserve and patient selection.
Zhao et al. [13]	Meta-analysis of randomized controlled trials	Combined MSC and PRP therapy	MSCs, PRP, or conventional treatment	Multiple randomized trials	Variable	Pain and functional outcomes	Combination therapy demonstrated potential clinical benefit, although consistent superiority over single biologic interventions was not established.
Tang et al. [14]	Meta-analysis	PRP	HA	1,281 participants	1-12 months; some studies extended to 18 months	VAS and WOMAC	PRP generally provided more durable pain relief and functional improvement than HA, particularly during intermediate follow-up.
Lamo-Espinosa et al. [15]	Multicenter randomized phase I/II trial	Autologous BM-MSCs at different cellular doses	HA	30 participants enrolled; 25 completed follow-up	48 months	Pain and functional outcomes	BM-MSC treatment demonstrated sustained clinical improvement, although the optimal cellular dose remains incompletely defined.
Matas et al. [16]	Randomized phase I/II trial	Repeated or single-dose UC-MSC therapy	HA	26 participants	12 months	VAS and WOMAC	Repeated UC-MSC administration produced more durable improvement than a single dose, supporting the potential importance of treatment scheduling.
Lee et al. [17]	Randomized placebo-controlled phase IIb trial	Autologous AD-MSCs	Placebo	24 participants	6 months	Pain, function, and imaging outcomes	AD-MSC therapy produced encouraging symptomatic outcomes and preliminary structural signals, although definitive cartilage restoration was not established.
Lu et al. [18]	Randomized, double-blind phase IIb trial	Autologous adipose-derived mesenchymal progenitor cells	HA	53 randomized; 52 treated; 47 completed follow-up	12 months	WOMAC and quantitative MRI	Clinical improvement was accompanied by favorable imaging findings, although the relationship between structural and symptomatic response remained uncertain.
Anz et al. [19]	Prospective randomized trial	BMAC	PRP	90 participants enrolled; 84 completed follow-up	12 months	Pain and patient-reported function	BMAC and PRP produced comparable clinical improvement, suggesting that increased biological complexity does not necessarily translate into superior outcomes.
Campbell et al. [20]	Systematic review of overlapping meta-analyses	PRP	HA, corticosteroids, placebo, or other comparators	Multiple overlapping meta-analyses	Variable	Pain and function	The evidence generally favored PRP for symptomatic improvement, but conclusions were limited by overlap, heterogeneity, and variable methodological quality.
Berrigan et al. [21]	Systematic review	PRP categorized by platelet dose	Variable controls	Multiple clinical studies	Variable	Pain and functional outcomes	Higher platelet dose may be associated with greater clinical response, although standardized dose thresholds remain unavailable.
Qiao et al. [22]	Clinical investigation	Autologous PRP	Baseline or conventional comparison	NR	Short-term follow-up	Pain and inflammatory biomarkers	PRP was associated with symptomatic improvement and reductions in inflammatory markers, supporting a biologically plausible immunomodulatory effect.
Tangkanjanavelukul et al. [23]	Prospective randomized trial with blinded endpoint assessment	AD-MSC therapy	HA	47 participants	6 months	Clinical and imaging outcomes	AD-MSC therapy demonstrated symptomatic benefit and preliminary cartilage-related imaging changes in early disease.
Romandini et al. [24]	Double-blind randomized controlled trial	Leukocyte-rich versus leukocyte-poor PRP	Alternative PRP formulations	NR	NR	Pain, function, and safety	Leukocyte concentration alone did not appear to determine clinical efficacy or safety, underscoring the multidimensional nature of PRP composition.
McLarnon and Heron [25]	Systematic review and meta-analysis	PRP	Intra-articular corticosteroids	648 participants	3-12 months	Pain and function	PRP generally provided more durable symptomatic improvement than corticosteroids, whose effect was more transient.
Han et al. [26]	Meta-analysis	PRP	HA	1,314 participants	1-12 months; one study extended to 18 months	VAS and WOMAC	PRP was associated with more sustained pain relief and functional improvement than HA, particularly at intermediate follow-up.
Di et al. [27]	Systematic review of randomized controlled trials	PRP	HA	908 participants	6 weeks-12 months	Pain and function	PRP generally demonstrated superior symptomatic outcomes, although variability in injection protocols limited direct comparison.
Montañez-Heredia et al. [28]	Randomized clinical trial	PRP	HA	53 participants	6 months	Pain and functional outcomes	PRP and HA produced clinical improvement, with findings supporting PRP as a reasonable nonsurgical option in selected patients.
Bennell et al. [29]	Randomized placebo-controlled trial	PRP	Saline placebo	NR	12 months	Pain and medial tibial cartilage volume	PRP did not significantly improve pain or cartilage volume compared with placebo, highlighting variability in treatment-specific efficacy.
Ghorbani et al. [30]	Randomized clinical trial	PRP	HA	NR	Short-term follow-up	Pain and function	Both interventions improved symptoms, while comparative differences remained dependent on the outcome and assessment period.
Sun et al. [31]	Clinical study combining biologic and corrective treatment	AD-MSCs with high tibial osteotomy	High tibial osteotomy alone	45 participants	48 months overall; principal comparative outcomes reported through 24 months	Clinical, functional, imaging, and cellular outcomes	Outcomes were influenced by cellular quality and biomechanical correction, illustrating the interaction between biological and mechanical treatment.
Pabinger et al. [32]	Long-term clinical cohort	BMAC	No concurrent active comparator	37 knees	4 years	Pain and function	Sustained symptomatic improvement was reported in advanced KOA, although the uncontrolled design limits causal inference.
Baria et al. [33]	Randomized controlled trial	MFAT	PRP	58 participants	12 months	KOOS and VAS	MFAT and PRP produced comparable improvement, suggesting that the greater procedural complexity of MFAT may not consistently provide additional clinical benefit.
Gobbi et al. [34]	Prospective randomized clinical trial	MFAT	Leukocyte-poor PRP combined with HA	50 participants	24 months	Pain and patient-reported function	Both strategies produced sustained and comparable clinical improvement during two-year follow-up.
Delgado-Enciso et al. [35]	Phase I/II randomized controlled trial	Cell-free biologic formulation	Active or conventional control	24 participants	12 months	Pain, function, and structural outcomes	The intervention demonstrated preliminary clinical potential, but confirmatory evidence from larger trials remains necessary.
Totlis et al. [36]	Prospective comparative study	SVF combined with PRP	HA	NR	NR	Pain and functional outcomes	The combined cellular strategy produced favorable clinical outcomes, although its incremental benefit and reproducibility require further evaluation.

Biological Basis of Orthobiologic Therapies

Osteoarthritis was traditionally regarded as a predominantly degenerative disorder driven by aging, mechanical overload, and progressive loss of articular cartilage. It is now understood, however, as a complex disease of the entire joint, involving coordinated pathological changes in the cartilage, synovium, subchondral bone, menisci, ligaments, and periarticular tissues. Persistent low-grade inflammation contributes actively to disease progression by promoting extracellular matrix degradation, cellular senescence, oxidative stress, and impaired tissue repair. In particular, synovial inflammation enhances the release of pro-inflammatory cytokines, including tumor necrosis factor-alpha (TNF-α), together with matrix metalloproteinases (MMPs), thereby sustaining a catabolic intra-articular microenvironment that disrupts cartilage homeostasis and accelerates structural deterioration. Accordingly, osteoarthritis is no longer interpreted solely as the consequence of mechanical wear but rather as a multifactorial biological disorder in which inflammation, altered tissue crosstalk, and insufficient endogenous repair interact throughout the course of the disease [2,5,10-13].

This broader pathophysiological framework provides the biological rationale for the use of orthobiologic therapies in regenerative musculoskeletal medicine. Orthobiologics are biologically derived products intended to modulate the diseased joint environment by restoring, at least partially, the balance between anabolic and catabolic signaling rather than acting exclusively through transient symptom suppression [1,2,14-20]. PRP, MSCs, BMAC, and adipose-derived cellular products exert their effects predominantly through immunomodulatory, trophic, and paracrine mechanisms [1,2,14-18]. PRP delivers a concentrated milieu of bioactive mediators, including platelet-derived growth factor (PDGF), transforming growth factor-beta (TGF-β), vascular endothelial growth factor (VEGF), insulin-like growth factor-1 (IGF-1), and fibroblast growth factor (FGF), which may influence cellular proliferation, extracellular matrix synthesis, angiogenesis, and tissue-repair responses [1,10,14,21,22]. MSC-based therapies act principally through the secretion of cytokines, extracellular vesicles, growth factors, and anti-inflammatory mediators that regulate macrophage polarization, attenuate inflammatory signaling, and support endogenous reparative processes without directly replacing damaged cartilage [1,2,14-20].

The preservation of articular cartilage depends on a tightly regulated state of joint homeostasis in which chondrocytes continuously balance extracellular matrix synthesis and degradation. In osteoarthritis, chronic inflammatory and mechanical stress shifts this equilibrium toward catabolism, resulting in collagen breakdown, proteoglycan depletion, oxidative injury, and progressive loss of cellular reparative capacity [2,5,10-13]. Orthobiologic therapies seek to modify this unfavorable environment by reducing inflammatory signaling, supporting anabolic activity, and preserving the function of residual joint tissues [1,2,14-20]. Importantly, the available evidence suggests that their clinical effects are more plausibly explained by modulation of the intra-articular microenvironment than by complete restoration of normal hyaline cartilage. Improvements in pain and function, therefore, support the biological relevance of these interventions but should not be interpreted as definitive proof of structural regeneration or disease modification [1,2,14,15]. Overall, the recognition of osteoarthritis as a biologically active whole-joint disorder provides the central justification for regenerative strategies aimed at maintaining tissue homeostasis and preserving native joint function, while further research remains necessary to identify the products, protocols, and patient phenotypes most likely to derive meaningful benefit [16,17,23].

PRP

PRP is the most extensively investigated orthobiologic intervention for KOA and currently represents the biologic therapy supported by the largest body of clinical evidence. PRP is an autologous blood-derived product obtained through centrifugation techniques that increase platelet concentration while retaining a complex mixture of bioactive proteins involved in inflammation, tissue repair, and cellular signaling [1,10,14,21,22]. Following activation, platelets release mediators such as PDGF, TGF-β, VEGF, IGF-1, FGF, and epidermal growth factor, which may influence chondrocyte metabolism, extracellular matrix synthesis, angiogenesis, and tissue-repair responses [1,10,14,21,22]. Beyond growth-factor delivery, PRP may attenuate intra-articular inflammation by reducing the expression of interleukin-1β, TNF-α, and MMPs, thereby promoting a more favorable anabolic and immunomodulatory environment [10,22]. Its therapeutic rationale therefore lies primarily in modulation of the pathological joint microenvironment rather than in direct restoration of normal hyaline cartilage.

Despite its widespread use, PRP remains a biologically heterogeneous intervention. Meaningful differences exist in blood collection, centrifugation systems, platelet concentration, leukocyte content, activation methods, injected volume, number of applications, and intervals between injections, making apparently similar PRP protocols difficult to compare [3,10,21]. The distinction between leukocyte-poor PRP and leukocyte-rich PRP has received particular attention because leukocyte concentration may alter the inflammatory profile of the final product. Although leukocyte-poor formulations have often been favored on the basis of a theoretically lower pro-inflammatory response, recent randomized evidence suggests that leukocyte content alone may not determine either safety or clinical efficacy [24]. Treatment response is more likely influenced by the interaction of multiple product- and patient-level characteristics, including platelet dose, preparation quality, disease severity, baseline inflammatory status, and administration schedule [3,10,21]. This multidimensional heterogeneity remains one of the principal barriers to reproducibility and to the development of universally accepted preparation and dosing standards.

The overall clinical evidence supports PRP as an effective option for symptomatic mild-to-moderate KOA, particularly for improving pain and patient-reported function during short- and intermediate-term follow-up [1,10,14,25-30]. Systematic reviews and comparative trials have frequently reported greater or more durable symptomatic benefit than hyaluronic acid, corticosteroids, or placebo, although the magnitude of effect varies according to comparator, formulation, injection protocol, and patient phenotype [1,14,25-30]. Emerging evidence suggests that repeated administrations and higher platelet concentrations may improve clinical response, but the optimal platelet dose, number of injections, and interval between treatments remain uncertain [3,21]. Outcomes also appear more favorable in patients with early-to-moderate disease, in whom viable cartilage and a less advanced biomechanical and inflammatory burden may permit a more meaningful response to biologic modulation [1,10,14,28,29]. Accordingly, PRP currently occupies the most extensive and comparatively mature clinical evidence-based position among orthobiologic therapies for KOA, although further standardization of product characterization, treatment protocols, outcome reporting, and patient selection is necessary before its effects can be reproduced consistently across clinical settings [1,3,10,14,21,22,24,29].

MSC-Based Therapies

MSC-based therapies represent one of the most promising regenerative strategies for KOA because of their capacity to modulate inflammation, support tissue homeostasis, and potentially influence structural disease progression. Although MSCs possess multilineage differentiation potential, their therapeutic effects appear to be mediated predominantly through paracrine and immunomodulatory signaling rather than direct replacement of damaged cartilage [2,15,16,31]. Through the release of cytokines, growth factors, extracellular vesicles, and other bioactive mediators, MSCs may attenuate synovial inflammation, suppress catabolic pathways, regulate immune-cell activity, and support endogenous reparative processes within the joint [2,15,16,31]. Their contemporary role in regenerative medicine is therefore better understood as biological modulation of the intra-articular environment than as simple cellular substitution.

Several cellular sources have been investigated, including bone marrow-derived MSCs, adipose-derived MSCs, and umbilical cord-derived MSCs, each with distinct biological, logistical, and therapeutic characteristics. Bone marrow-derived MSCs remain among the most extensively studied and have been associated with sustained improvements in pain and function, although the relationship between administered cell dose and clinical response remains incompletely defined [15]. Adipose-derived MSCs offer practical advantages related to relatively high cellular yield and accessible harvesting and have demonstrated encouraging symptomatic and functional outcomes, together with preliminary signals of structural preservation in selected studies [17,18,23,31]. Umbilical cord-derived MSCs have also attracted interest because of their proliferative capacity, allogeneic availability, and relatively low immunogenicity; repeated administration has been associated with more durable clinical improvement than single-dose treatment in early-phase trials [16]. Nevertheless, marked variability in tissue source, harvesting technique, cell isolation and expansion, processing conditions, viability, dose, and administration protocol continues to limit meaningful comparisons across studies.

The available clinical evidence suggests that MSC-based therapies may improve pain, physical function, and quality of life, particularly in patients with early-to-moderate KOA [2,15,31]. However, the magnitude and durability of benefit vary across products, study designs, and patient populations. Some investigations have reported favorable changes in cartilage morphology, defect stabilization, or imaging-based markers of disease progression [17,18,23], but structural regeneration has not been demonstrated consistently, and radiologic changes do not invariably correlate with symptomatic improvement. Accordingly, MSC-based therapies should currently be regarded as promising joint-preservation interventions with substantial biological plausibility but not as established means of restoring normal articular cartilage. Their broader clinical implementation will depend on higher-quality long-term evidence and greater standardization of cell source, manufacturing and processing methods, dose characterization, treatment schedules, and patient-selection criteria [2,15-18,23,31].

Emerging Orthobiologic Therapies

Beyond PRP and MSC-based therapies, a growing range of orthobiologic strategies has been developed to expand the possibilities of biologically oriented joint preservation in KOA. These approaches include BMAC, MFAT, SVF, cell-free biologic formulations, hybrid cellular products, and combination therapies designed to exploit complementary anti-inflammatory, trophic, and reparative mechanisms [19,28,32-36]. Although the evidence base remains less mature than that supporting PRP, these interventions reflect the continued evolution of regenerative medicine toward more individualized treatment strategies adapted to the biological and structural characteristics of the affected joint [19,28,32-36].

BMAC is among the most extensively studied of these emerging therapies. Unlike culture-expanded or isolated MSC products, BMAC is a heterogeneous autologous preparation containing mesenchymal progenitor cells, hematopoietic cells, platelets, cytokines, and growth factors within a minimally manipulated biologic matrix [16,22]. This composite profile may support immunomodulation, angiogenic signaling, and endogenous tissue-repair responses, although the relative contribution of each component remains incompletely defined. Clinical studies have reported improvements in pain and physical function, with some cohorts describing sustained symptomatic benefit over extended follow-up periods [19,32]. MFAT similarly preserves elements of the native adipose stromal niche, including pericytes, stromal cells, extracellular matrix components, and anti-inflammatory mediators. Randomized comparisons of MFAT with PRP or leukocyte-poor PRP combined with hyaluronic acid have generally demonstrated comparable improvements in patient-reported outcomes, suggesting that biologically distinct products may achieve similar symptomatic effects despite marked differences in cellular composition and processing [33,34].

Additional regenerative strategies include SVF, cell-free formulations, and hybrid approaches combining cellular products with PRP, hyaluronic acid, or biomechanical correction [31,35,36]. These interventions are intended to address several components of osteoarthritis simultaneously, including synovial inflammation, impaired tissue homeostasis, and abnormal mechanical loading. However, their incremental clinical value remains uncertain, and most are still supported by small studies, heterogeneous preparation protocols, limited comparator data, and relatively short follow-up [19,28,32-36]. Consequently, these therapies should currently be regarded as investigational or selectively applicable rather than routinely established options. Their future integration into evidence-based practice will depend on standardized manufacturing and characterization, adequately powered multicenter randomized trials, longer-term structural and clinical assessment, and clearer identification of the patient phenotypes most likely to benefit [19,28,32-36]. A comparative overview of the biological characteristics, clinical evidence, principal advantages, limitations, and potential roles of the major orthobiologic therapies is provided in Table 4.

**Table 4:** Comparative characteristics of orthobiologic therapies for KOA. References were selected as representative sources supporting the biological rationale, clinical outcomes, safety profile, and principal limitations summarized for each therapeutic category. AD-MSC, adipose-derived mesenchymal stromal cell; BMAC, bone marrow aspirate concentrate; BM-MSC, bone marrow-derived mesenchymal stromal cell; HA, hyaluronic acid; KOA, knee osteoarthritis; MFAT, microfragmented adipose tissue; MSC, mesenchymal stromal cell; PRP, platelet-rich plasma; SVF, stromal vascular fraction; UC-MSC, umbilical cord-derived mesenchymal stromal cell

Therapy	Biological composition	Principal mechanism of action	Clinical evidence	Main advantages	Main limitations	Potential clinical role	References
PRP	Autologous platelet concentrate enriched with growth factors and bioactive proteins	Immunomodulation, attenuation of inflammatory signaling, support of anabolic activity, extracellular matrix homeostasis, and tissue-repair responses	Supported by the most consistent evidence for improvement in pain and patient-reported function, particularly during short- and intermediate-term follow-up; structural benefit remains uncertain	Widely available, minimally invasive, autologous, and associated with a favorable safety profile	Marked variability in platelet concentration, leukocyte content, activation methods, injected volume, number of applications, and duration of response	Reasonable first-line orthobiologic option for selected patients with symptomatic early-to-moderate KOA	[1,3,10,14,21,24-30]
BM-MSCs	Culture-expanded or isolated MSCs obtained from bone marrow	Paracrine signaling, immunomodulation, macrophage polarization, trophic support, and restoration of tissue homeostasis	Associated with encouraging improvements in pain and function, with preliminary structural findings in selected studies	Strong biological rationale and relatively extensive clinical experience among cellular therapies	Invasive harvesting, variability in expansion and processing methods, uncertain optimal cell dose, and limited long-term standardization	Cellular option for selected patients with early-to-moderate KOA and preserved biological and structural reserve	[2,5,11,12,15]
AD-MSCs	MSCs isolated or expanded from adipose tissue	Anti-inflammatory, immunomodulatory, and trophic paracrine effects supporting cartilage homeostasis and endogenous repair	Favorable symptomatic and functional outcomes, with preliminary imaging signals of structural preservation in selected investigations	Relatively high cellular yield and accessible tissue source	Heterogeneous isolation and processing techniques, variable cellular products, and limited protocol standardization	Alternative cellular therapy for carefully selected patients with residual joint integrity	[2,17,18,23,31]
UC-MSCs	Allogeneic MSCs derived from umbilical cord tissue	Immunomodulatory and paracrine signaling with high proliferative capacity and relatively low immunogenicity	Early clinical evidence suggests meaningful symptomatic improvement; repeated dosing may provide more durable benefit than a single administration	Allogeneic availability, high proliferative potential, and no autologous harvesting procedure	Limited long-term evidence, manufacturing variability, regulatory complexity, and restricted comparative data	Emerging cellular option that requires further clinical validation before routine implementation	[2,12,16]
BMAC	Heterogeneous autologous concentrate containing mesenchymal progenitor cells, hematopoietic cells, platelets, cytokines, and growth factors	Combined cellular and humoral activity supporting immunomodulation, angiogenic signaling, and endogenous repair	Promising improvements in pain and function, including sustained benefit in selected cohorts; comparative superiority remains uncertain	Single-stage autologous procedure containing multiple biologically active components	Variability in aspiration technique, processing platform, cellular yield, and final composition	Joint-preservation option for selected patients when procedural complexity and evidence limitations are carefully considered	[6,7,19,32]
MFAT	Minimally manipulated adipose tissue preserving stromal cells, pericytes, extracellular matrix, and the native stromal niche	Anti-inflammatory and trophic signaling through preserved cellular and extracellular components	Randomized studies suggest symptomatic improvement comparable to PRP or PRP combined with HA	Preserves the native adipose microenvironment and provides a rich autologous cellular matrix	More invasive harvesting than PRP, greater procedural complexity, and limited comparative evidence	Alternative autologous orthobiologic option in selected patients, without established superiority over simpler interventions	[33,34]
SVF	Heterogeneous adipose-derived cellular fraction containing stromal, vascular, immune, and progenitor-cell populations	Multicellular immunomodulatory, trophic, and reparative signaling	Preliminary comparative evidence suggests improvements in pain and function, but the evidence base remains limited	Broad cellular composition and potential for multimodal biological activity	Major heterogeneity in isolation methods, uncertain product characterization, and regulatory concerns	Investigational therapy requiring standardized manufacturing and larger comparative trials	[36]
Cell-free biologic formulations	Acellular preparations containing bioactive proteins, signaling molecules, or secreted factors	Modulation of inflammation and tissue repair without direct cellular administration	Early-phase evidence suggests potential symptomatic and structural benefit, although confirmation is required	Potentially improved reproducibility and reduced cellular-manufacturing complexity	Very limited evidence, uncertain composition, lack of standardization, and developmental regulatory status	Emerging investigational strategy rather than an established clinical option	[35]
Combination biologic therapies	Combinations such as PRP with MSCs, PRP with HA, or biologic therapy with corrective procedures	Complementary modulation of inflammation, lubrication, trophic signaling, tissue homeostasis, and biomechanical loading	Some studies report favorable or sustained clinical outcomes, but consistent additive benefit over single therapies has not been established	May address multiple biological or mechanical components of KOA simultaneously	Heterogeneous combinations, limited head-to-head evidence, and absence of standardized protocols	Potential component of future phenotype-guided treatment pathways rather than a universally superior strategy	[13,31,34]

Clinical Outcomes and Patient-Reported Outcomes

Across the available literature, orthobiologic therapies have been consistently associated with clinically meaningful improvements in pain, physical function, and patient-reported outcomes in individuals with KOA. Among the available interventions, PRP is supported by the most consistent body of evidence, with multiple randomized trials and systematic reviews demonstrating greater symptomatic improvement than hyaluronic acid, intra-articular corticosteroids, and placebo across commonly used outcome measures, including the VAS, the WOMAC, the KOOS, and the International Knee Documentation Committee score [1,2,14,25,26,37]. BMAC has likewise demonstrated favorable clinical outcomes; however, the available evidence remains comparatively limited and methodologically heterogeneous. Overall, PRP appears to provide the most reproducible short- and intermediate-term improvements in pain and function, with therapeutic benefits most frequently reported during the first three to 12 months following treatment [14,25,26].

MSC-based therapies have shown similarly encouraging clinical results, particularly with respect to the durability of symptomatic improvement. Several studies have reported sustained reductions in pain and improvements in physical function extending beyond one year, with selected cohorts demonstrating persistent benefit during longer-term follow-up [2,15,31]. Emerging evidence also suggests that treatment timing and administration strategy may influence clinical response. Patients with early-to-moderate disease, preserved joint architecture, and lower inflammatory burden appear more likely to achieve favorable outcomes, while repeated administration protocols have been associated with enhanced and more sustained symptomatic improvement in selected studies [3,16,27]. Nevertheless, substantial heterogeneity in biologic preparation, dosing, treatment schedules, and patient selection continues to limit direct comparison across interventions and prevents definitive conclusions regarding optimal therapeutic protocols.

Beyond symptomatic improvement, increasing attention has focused on the potential structural effects of orthobiologic therapies. Although PRP primarily demonstrates anti-inflammatory and symptom-modifying properties, MSC-based therapies and BMAC have shown preliminary evidence of structural preservation in selected investigations, including stabilization of cartilage defects, reduced synovial inflammation, preservation of cartilage volume, and attenuation of radiographic progression [15,17,18,23,31]. These findings, however, have not been consistently reproduced across studies. Imaging outcomes remain heterogeneous, and improvements observed on magnetic resonance imaging do not consistently parallel changes in pain or physical function, underscoring the continuing uncertainty regarding the relationship between structural modification and clinically meaningful benefit [2,16]. Consequently, current evidence remains insufficient to conclude that orthobiologic therapies consistently modify the natural history of KOA.

Overall, the available literature supports a favorable safety profile for the principal orthobiologic therapies. Reported adverse events are generally mild, self-limiting, and transient, most commonly consisting of post-injection pain, localized swelling, stiffness, or short-lived synovitis, whereas serious treatment-related complications have been reported only rarely [2,14,25]. Taken together, the current evidence supports the use of orthobiologic interventions as safe therapeutic options capable of improving pain, function, and quality of life in appropriately selected patients. Among the available biologic therapies, PRP remains supported by the most consistent evidence for symptomatic improvement, whereas cellular therapies demonstrate the greatest biological potential for sustained clinical benefit. Nevertheless, further high-quality comparative studies with standardized protocols and long-term follow-up are required before definitive conclusions regarding disease modification or comparative superiority can be established [1,2,14].

Patient Selection and Clinical Considerations

Patient selection is a central determinant of therapeutic response to orthobiologic interventions in KOA. Across the available literature, disease severity emerges as one of the most consistent predictors of outcome, with patients presenting with early-to-moderate osteoarthritis generally achieving greater improvements than those with advanced joint degeneration [2,15,23,28,32-35]. Studies evaluating PRP, MSC-based therapies, and other regenerative approaches have reported more favorable pain, functional, and patient-reported outcomes among individuals with KL grades I-III, particularly grade II, whereas treatment efficacy tends to decline in the presence of extensive cartilage loss, subchondral remodeling, and severe structural compromise [15,23,28,34]. These findings suggest that orthobiologic therapies are most likely to produce clinically meaningful benefit when sufficient biological and structural reserve remains within the joint to respond to anti-inflammatory, immunomodulatory, and trophic signaling. Although selected patients with advanced disease may still experience symptomatic improvement, expectations regarding structural restoration, durable joint preservation, and disease modification should remain conservative in this population [3,15].

Additional patient-level characteristics may further influence treatment response, although the evidence supporting these associations is less consistent than that related to disease stage. Younger age, lower BMI, reduced inflammatory burden, and less severe baseline degeneration have been proposed as potential predictors of improved outcomes, but these relationships have not been uniformly demonstrated across studies [16,34,36]. The inflammatory phenotype of the joint may also be clinically relevant, as interventions that modulate synovitis and restore tissue homeostasis may be more effective in patients with a biologically active but still structurally salvageable joint environment [2,23,36]. Treatment-related variables should likewise be considered. Repeated administrations of PRP or MSC-based therapies have been associated with more sustained clinical responses than single-dose protocols in selected studies, while optimization of cellular dose, injection interval, and overall treatment schedule may further influence efficacy [3,15,16]. These observations reinforce the need to individualize treatment according to both patient biology and intervention characteristics rather than relying on a uniform product-centered approach.

Biomechanical factors are equally important because biological modulation alone is unlikely to overcome persistent abnormal joint loading. Several studies have excluded patients with marked varus or valgus deformity or have combined orthobiologic interventions with corrective procedures, reflecting concern that severe malalignment may compromise tissue homeostasis and limit therapeutic response [3,15,31,34]. The most favorable candidate is therefore likely to be a patient with symptomatic early-to-moderate KOA, preserved or correctable alignment, residual cartilage and joint architecture, and a clinical phenotype in which inflammation and functional limitation remain potentially modifiable. Conversely, treatment failure is more likely in end-stage degeneration, severe malalignment, extensive subchondral disease, or when an orthobiologic intervention is expected to compensate for major mechanical pathology in isolation. Collectively, the evidence supports a precision-medicine framework in which disease stage, inflammatory profile, biomechanical environment, and patient-specific characteristics may be as influential as the orthobiologic product itself in determining clinical success [2,15,23,31,34]. The principal clinical factors influencing patient selection and therapeutic decision-making for orthobiologic interventions are summarized in Table 5.

**Table 5:** Clinical considerations for patient selection and therapeutic decision-making in KOA. Clinical considerations presented in this table are derived from the collective interpretation of the available evidence and should not be regarded as definitive treatment indications. Therapeutic selection should integrate disease severity, patient-specific biological and biomechanical characteristics, treatment objectives, and shared clinical decision-making. The current evidence supports orthobiologic therapies primarily as joint-preserving adjuncts rather than universal disease-modifying interventions. This interpretation is consistent with contemporary evidence reviews and recent expert consensus statements on orthobiologic use in KOA. KL, Kellgren-Lawrence; KOA, knee osteoarthritis; MSC, mesenchymal stromal cell; PRP, platelet-rich plasma

Clinical factor	Influence on therapeutic response	Clinical implication	Supporting evidence
KL grade I-II	Highest probability of favorable response due to greater biological and structural reserve	Consider PRP or selected cellular therapies before advanced structural deterioration develops	[3,7,12,15-20]
KL grade III	Moderate clinical response with greater variability	Individualized treatment according to symptoms, imaging findings, and remaining joint integrity	[3,7,12,15-20]
KL grade IV	Lower probability of sustained benefit because of extensive structural damage	Orthobiologics may provide symptomatic improvement but should not replace appropriately indicated arthroplasty	[7,12,19,20]
Preserved joint alignment	Associated with improved biological response	Corrective biologic treatment may be considered when biomechanical conditions are favorable	[6,7,12,18,31]
Severe varus or valgus malalignment	Persistent abnormal loading may limit therapeutic efficacy	Mechanical correction should be considered before or together with biologic intervention	[6,7,12,18,31]
Younger age and lower biological burden	May enhance regenerative and immunomodulatory capacity	Greater likelihood of sustained symptomatic improvement in appropriately selected patients	[3,10,18,21]
Lower BMI	Reduced mechanical overload and inflammatory burden may improve outcomes	Weight optimization should accompany biologic treatment whenever appropriate	[3,10,18,21]
Active synovial inflammation	May represent a biologically responsive phenotype for immunomodulatory therapies	Clinical and imaging assessment of inflammatory activity may assist patient selection	[3,15,21,23]
Repeated treatment protocols	Multiple administrations may prolong clinical benefit compared with single injections	Treatment schedule should be individualized according to biologic product and clinical response	[3,10,12,16]
Advanced structural degeneration	Limited potential for meaningful biological modulation	Expectations should focus on symptom relief rather than cartilage regeneration or disease reversal	[3,7,12,19,20]
Multimodal management	Orthobiologics perform best when integrated with rehabilitation and biomechanical optimization	Combine biologic therapy with exercise, weight management, physical therapy, and corrective procedures when indicated	[6,7,18,23,31]

Discussion

The present review supports a transition in KOA management from isolated symptom control toward biologically informed joint preservation. Osteoarthritis is increasingly understood as a whole-joint disorder in which cartilage degeneration, synovial inflammation, subchondral bone remodeling, biomechanical dysfunction, and impaired endogenous repair interact throughout disease progression. This framework helps explain why conventional interventions may improve pain without necessarily altering the biological processes that sustain tissue deterioration. The contemporary relevance of orthobiologic therapies, therefore, lies not in their ability to reliably regenerate normal hyaline cartilage but in their potential to attenuate inflammatory amplification, modulate the intra-articular microenvironment, and preserve the function of residual joint tissues [5,10-12,22]. Their clinical role should consequently be interpreted within a comprehensive joint-preservation strategy rather than as that of an isolated injectable treatment.

The biological effects of orthobiologic therapies appear to be mediated predominantly through immunomodulatory, trophic, and paracrine signaling. Platelet-derived preparations deliver concentrated growth factors and bioactive proteins capable of influencing inflammatory pathways, chondrocyte metabolism, extracellular matrix turnover, and tissue-repair responses [10,14,22]. MSC-based therapies similarly act through the secretion of cytokines, extracellular vesicles, and other mediators that may regulate macrophage polarization, synovial inflammation, apoptosis, and tissue homeostasis [2,5,11-13]. These mechanisms may create a less catabolic environment in which remaining cartilage and periarticular tissues retain greater functional capacity. Importantly, however, the available evidence supports biological modulation more consistently than direct cellular replacement or complete structural restoration. Orthobiologic therapies may therefore support a biologically responsive joint, but they cannot be expected to reverse advanced malalignment, extensive cartilage loss, or established mechanical failure.

The most reproducible clinical effects of orthobiologic interventions are improvements in pain and patient-reported physical function, particularly during short- and intermediate-term follow-up. Meta-analyses frequently indicate that PRP may provide greater or more durable symptomatic improvement than hyaluronic acid or intra-articular corticosteroids, although the magnitude of benefit varies according to formulation, comparator, administration protocol, and patient phenotype [2,5,10,13,14,25]. MSC-based therapies and combined biologic approaches have likewise demonstrated encouraging improvements in pain, function, and quality of life [3,10,13,14,25]. Nevertheless, neutral findings from placebo-controlled trials, including the RESTORE trial, illustrate that therapeutic effects are not uniform across products or populations [29]. This variability likely reflects both genuine biological differences between preparations and the methodological heterogeneity that characterizes the current literature.

Evidence supporting structural preservation or disease modification remains substantially less certain than evidence for symptomatic improvement. Selected studies have reported favorable imaging findings, cartilage-defect stabilization, reduced synovial inflammation, or attenuation of radiographic progression; however, these observations have not been reproduced consistently, and structural changes do not invariably correlate with improvements in pain or function [2,17,18,23]. Similarly, the capacity of orthobiologic therapies to delay total knee arthroplasty has not been established with sufficient certainty [3,15,35]. Clinical improvement should therefore not be interpreted as proof of cartilage regeneration or alteration of the natural history of osteoarthritis. At present, these interventions are more appropriately regarded as adjunctive treatments that may create a therapeutic window for rehabilitation, exercise, weight optimization, and biomechanical correction rather than as validated substitutes for definitive surgical management.

Patient selection appears to be one of the most important determinants of therapeutic success. Patients with early-to-moderate KOA, particularly those with KL grades II-III, generally demonstrate more favorable outcomes than individuals with advanced structural degeneration [2,15,23,28,32-35]. This pattern is biologically plausible because residual cartilage integrity, viable chondrocyte populations, and a less hostile intra-articular environment may permit anti-inflammatory and trophic mechanisms to exert a clinically meaningful effect. Lower BMI, reduced inflammatory burden, preserved alignment, and appropriate activity levels may further support treatment response by limiting the metabolic and biomechanical forces that perpetuate disease progression [2,3,15,16,23,31,34,36]. These observations suggest that the biological and mechanical context of the recipient joint may be as influential as the orthobiologic product itself. Although selected patients with advanced osteoarthritis may still experience symptomatic improvement, expectations regarding structural restoration, durable joint preservation, and avoidance of surgery should remain conservative [3,15,35].

Biomechanical factors must therefore remain central to treatment planning. Persistent varus or valgus malalignment, instability, meniscal insufficiency, and abnormal joint loading may continue to promote tissue degeneration despite successful biological modulation [3,15,31,34]. Orthobiologic interventions should not be expected to compensate for major structural pathology in isolation. Their greatest clinical value may lie within individualized, multimodal pathways that integrate rehabilitation, weight management, activity modification, mechanical unloading, and corrective procedures when indicated. Within this framework, biologic therapy may facilitate symptom control and functional improvement while preserving the native joint for a clinically meaningful period. This approach positions orthobiologics as one component of comprehensive musculoskeletal care rather than as an alternative to all established nonsurgical or surgical treatments.

The translation of orthobiologic therapies into routine practice is constrained by substantial variability in product composition and treatment protocols. PRP preparations differ in platelet concentration, leukocyte content, activation methods, injected volume, and number of applications, whereas MSC-based therapies vary according to tissue source, isolation and expansion techniques, processing conditions, and administered dose [2,3,14,15,26]. Comparable concerns apply to BMAC and adipose-derived products, for which harvesting methods, commercial processing systems, cellular yield, and final biological composition may influence both activity and clinical response [7,34]. Consequently, interventions classified under the same therapeutic category may represent fundamentally different biological products. This product-level heterogeneity limits reproducibility, complicates comparative effectiveness assessment, and restricts the development of clinically transferable treatment algorithms.

Separate from product variability, the certainty of the evidence is limited by small sample sizes, heterogeneous study designs, variable outcome measures, inconsistent definitions of treatment success, and insufficient long-term follow-up [2,5,7,15,18,28,32,35,38]. Publication bias and selective reporting may further contribute to an overly favorable perception of therapeutic efficacy. Structural outcomes are also evaluated using nonuniform imaging techniques and are frequently reported without standardized thresholds for clinically meaningful change. These methodological limitations make it difficult to determine whether observed benefits reflect product-specific effects, contextual placebo responses, differences in patient selection, or variation in concomitant care. Future investigations should therefore prioritize harmonized reporting standards, predefined clinically important outcomes, transparent biologic characterization, and longitudinal evaluation of symptomatic, structural, and arthroplasty-related endpoints.

The next phase of biologic joint preservation will likely depend on a transition from product-centered treatment toward phenotype-guided care. Biomarker integration, validated imaging strategies, and predictive analytical models may help identify inflammatory, structural, or molecular phenotypes more likely to respond to specific interventions [2,23,31]. Cell-free formulations and other next-generation biologic approaches may eventually improve manufacturing reproducibility and allow more controlled therapeutic effects [2,23,35]. Artificial intelligence may also support treatment selection by integrating clinical, imaging, molecular, and biomechanical information; however, these applications remain developmental and should not yet be regarded as established clinical solutions. Large multicenter randomized trials, direct comparative studies, cost-effectiveness analyses, and standardized manufacturing protocols will be required before widespread implementation can be justified [1,14,28,35].

Overall, orthobiologic therapies represent a meaningful shift toward biologically driven and patient-centered joint preservation. Their most defensible contemporary role lies in improving pain and function in carefully selected patients while supporting broader strategies intended to maintain native-joint function. Current evidence does not establish these interventions as universal disease-modifying treatments or replacements for indicated surgery. Rather, their value depends on matching the biological intervention to disease stage, inflammatory phenotype, biomechanical environment, and individual clinical priorities. The principal contribution of this field is therefore not the identification of a universally superior injectable product but the emergence of a more precise and integrated model of musculoskeletal care in which biological modulation complements rehabilitation, mechanical optimization, and appropriately timed surgical intervention.

Current Limitations, Evidence Gaps, and Future Directions

Despite the encouraging clinical outcomes reported for PRP, MSC-based therapies, BMAC, and other regenerative approaches, several unresolved limitations continue to restrict their consistent integration into evidence-based clinical practice. The most pervasive challenge is the substantial methodological heterogeneity across studies, which complicates direct comparison between interventions and limits the interpretability of pooled or cross-study findings [1,2,14,25,26]. Variability extends across patient-selection criteria, disease severity, comparator groups, outcome measures, and follow-up duration. Equally important, substantial differences in product preparation and characterization create biologically distinct interventions within the same therapeutic category. PRP formulations vary in platelet concentration, leukocyte content, activation strategy, injected volume, and number of applications, whereas MSC-based therapies differ according to tissue source, isolation and expansion methods, processing conditions, cellular dose, and administration protocol [2,3,14,15,26]. Similar concerns apply to BMAC and adipose-derived products, for which harvesting technique, processing platform, cellular yield, and final biologic composition may materially influence clinical response [19,34]. This lack of standardization remains a principal barrier to reproducibility, comparative effectiveness assessment, and the development of clinically transferable treatment algorithms.

Important evidence gaps also persist regarding the durability and disease-modifying potential of orthobiologic therapies. Although improvements in pain and function are frequently reported, relatively few studies extend beyond two years, and long-term outcomes remain available only for selected cohorts [15,31]. The evidence supporting structural modification is even less definitive. Reports of cartilage-defect stabilization, increased cartilage volume, reduced synovial inflammation, or attenuation of radiographic progression remain heterogeneous, and imaging-based changes do not consistently correlate with patient-reported improvement [2,18,23,35]. This discordance underscores the need to distinguish symptomatic benefit from true alteration of disease biology. Future investigations should therefore incorporate standardized structural endpoints, validated imaging protocols, clinically meaningful thresholds of response, and longitudinal assessment of arthroplasty-free survival. Predictive biomarkers capable of identifying inflammatory, structural, or molecular phenotypes most likely to respond may further improve patient stratification and reduce empiric treatment selection [2,23,31].

Advancing the field will require adequately powered multicenter randomized controlled trials using rigorously characterized biologic products, standardized preparation methods, harmonized outcome measures, and extended follow-up [2,14,26]. Direct comparative studies are particularly needed to clarify relative effectiveness across PRP formulations, cell sources, BMAC, adipose-derived products, and combination strategies. Cost-effectiveness, regulatory consistency, manufacturing reproducibility, and real-world implementation should also be incorporated into future research agendas, because biological efficacy alone will not determine clinical adoption. Ultimately, the next phase of orthobiologic therapy in KOA will depend on the transition from product-centered intervention toward phenotype-guided treatment, integrating clinical presentation, imaging, inflammatory biology, and biomechanical context. Current evidence supports these therapies as promising components of joint-preservation care, but their routine implementation will require stronger comparative evidence, greater protocol standardization, and a more precise understanding of the patients most likely to derive meaningful and durable benefit.

## Conclusions

Current evidence indicates that orthobiologic therapies can provide clinically meaningful improvements in pain and physical function in appropriately selected patients with KOA, with PRP supported by the most consistent evidence for short- to intermediate-term symptomatic benefit. However, substantial heterogeneity in product composition, administration protocols, patient selection, and outcome assessment prevents the establishment of a universally superior intervention, while evidence of structural restoration or definitive disease modification remains insufficient. Orthobiologic therapies should therefore be incorporated as adjuncts within individualized, multimodal joint-preservation strategies that integrate rehabilitation, weight optimization, biomechanical correction, and appropriately timed surgical intervention. Their future clinical value will depend on rigorous product standardization, phenotype-guided patient selection, and high-quality comparative studies with long-term structural and patient-centered outcomes.

## References

[1] Auroux M, Debionne T, Mainbourg S, Chapurlat R (2025). Efficacy of intra-articular platelet-rich plasma compared with placebo in knee osteoarthritis: a systematic review and meta-analysis. Joint Bone Spine.

[2] Chen X, Zheng J, Yin L, Li Y, Liu H (2024). Transplantation of three mesenchymal stem cells for knee osteoarthritis, which cell and type are more beneficial? a systematic review and network meta-analysis. J Orthop Surg Res.

[3] Zhuang W, Li T, Li Y (2024). The varying clinical effectiveness of single, three and five intraarticular injections of platelet-rich plasma in knee osteoarthritis. J Orthop Surg Res.

[4] Bensa A, Previtali D, Sangiorgio A, Boffa A, Salerno M, Filardo G (2025). PRP injections for the treatment of knee osteoarthritis: the improvement is clinically significant and influenced by platelet concentration: a meta-analysis of randomized controlled trials. Am J Sports Med.

[5] Song Y, Zhang J, Xu H, Lin Z, Chang H, Liu W, Kong L (2020). Mesenchymal stem cells in knee osteoarthritis treatment: a systematic review and meta-analysis. J Orthop Translat.

[6] Han JH, Jung M, Chung K, Jung SH, Choi CH, Kim SH (2024). Bone marrow aspirate concentrate injections for the treatment of knee osteoarthritis: a systematic review of randomized controlled trials. Orthop J Sports Med.

[7] Dulic O, Rasovic P, Lalic I (2021). Bone marrow aspirate concentrate versus platelet rich plasma or hyaluronic acid for the treatment of knee osteoarthritis. Medicina (Kaunas).

[8] Bellamy N, Buchanan WW, Goldsmith CH, Campbell J, Stitt LW (1988). Validation study of WOMAC: a health status instrument for measuring clinically important patient relevant outcomes to antirheumatic drug therapy in patients with osteoarthritis of the hip or knee. J Rheumatol.

[9] Roos EM, Roos HP, Lohmander LS, Ekdahl C, Beynnon BD (1998). Knee Injury and Osteoarthritis Outcome Score (KOOS)--development of a self-administered outcome measure. J Orthop Sports Phys Ther.

[10] Hong M, Cheng C, Sun X, Yan Y, Zhang Q, Wang W, Guo W (2021). Efficacy and safety of intra-articular platelet-rich plasma in osteoarthritis knee: a systematic review and meta-analysis. Biomed Res Int.

[11] Di Matteo B, Vandenbulcke F, Vitale ND, Iacono F, Ashmore K, Marcacci M, Kon E (2019). Minimally manipulated mesenchymal stem cells for the treatment of knee osteoarthritis: a systematic review of clinical evidence. Stem Cells Int.

[12] Kyriakidis T, Pitsilos C, Iosifidou M, Tzaveas A, Gigis I, Ditsios K, Iosifidis M (2023). Stem cells for the treatment of early to moderate osteoarthritis of the knee: a systematic review. J Exp Orthop.

[13] Zhao J, Liang G, Han Y (2022). Combination of mesenchymal stem cells (MSCs) and platelet-rich plasma (PRP) in the treatment of knee osteoarthritis: a meta-analysis of randomised controlled trials. BMJ Open.

[14] Tang JZ, Nie MJ, Zhao JZ, Zhang GC, Zhang Q, Wang B (2020). Platelet-rich plasma versus hyaluronic acid in the treatment of knee osteoarthritis: a meta-analysis. J Orthop Surg Res.

[15] Lamo-Espinosa JM, Mora G, Blanco JF (2018). Intra-articular injection of two different doses of autologous bone marrow mesenchymal stem cells versus hyaluronic acid in the treatment of knee osteoarthritis: long-term follow up of a multicenter randomized controlled clinical trial (phase I/II). J Transl Med.

[16] Matas J, Orrego M, Amenabar D (2019). Umbilical cord-derived mesenchymal stromal cells (MSCs) for knee osteoarthritis: repeated MSC dosing is superior to a single msc dose and to hyaluronic acid in a controlled randomized phase I/II trial. Stem Cells Transl Med.

[17] Lee WS, Kim HJ, Kim KI, Kim GB, Jin W (2019). Intra-articular injection of autologous adipose tissue-derived mesenchymal stem cells for the treatment of knee osteoarthritis: a phase IIb, randomized, placebo-controlled clinical trial. Stem Cells Transl Med.

[18] Lu L, Dai C, Zhang Z (2019). Treatment of knee osteoarthritis with intra-articular injection of autologous adipose-derived mesenchymal progenitor cells: a prospective, randomized, double-blind, active-controlled, phase IIb clinical trial. Stem Cell Res Ther.

[19] Anz AW, Hubbard R, Rendos NK, Everts PA, Andrews JR, Hackel JG (2020). Bone marrow aspirate concentrate is equivalent to platelet-rich plasma for the treatment of knee osteoarthritis at 1 year: a prospective, randomized trial. Orthop J Sports Med.

[20] Campbell KA, Saltzman BM, Mascarenhas R, Khair MM, Verma NN, Bach BR Jr, Cole BJ (2015). Does intra-articular platelet-rich plasma injection provide clinically superior outcomes compared with other therapies in the treatment of knee osteoarthritis? A systematic review of overlapping meta-analyses. Arthroscopy.

[21] Berrigan WA, Bailowitz Z, Park A, Reddy A, Liu R, Lansdown D (2025). A greater platelet dose may yield better clinical outcomes for platelet-rich plasma in the treatment of knee osteoarthritis: a systematic review. Arthroscopy.

[22] Qiao J, Guo X, Zhang L, Zhao H, He X (2024). Autologous platelet rich plasma injection can be effective in the management of osteoarthritis of the knee: impact on IL-1 β, TNF-α, hs-CRP. J Orthop Surg Res.

[23] Tangkanjanavelukul P, Khuangsirikul S, Heebthamai D, Yamabhai M, Sumphanapai T, Khumtong N, Chotanaphuti T (2025). Cartilage regeneration potential in early osteoarthritis of the knee: a prospective, randomized, open, and blinded endpoint study comparing adipose-derived mesenchymal STEM cell (ADSC) therapy versus hyaluronic acid. Int J Mol Sci.

[24] Romandini I, Boffa A, Di Martino A (2024). Leukocytes do not influence the safety and efficacy of platelet-rich plasma injections for the treatment of knee osteoarthritis: a double-blind randomized controlled trial. Am J Sports Med.

[25] McLarnon M, Heron N (2021). Intra-articular platelet-rich plasma injections versus intra-articular corticosteroid injections for symptomatic management of knee osteoarthritis: systematic review and meta-analysis. BMC Musculoskelet Disord.

[26] Han Y, Huang H, Pan J (2019). Meta-analysis comparing platelet-rich plasma vs hyaluronic acid injection in patients with knee osteoarthritis. Pain Med.

[27] Di Y, Han C, Zhao L, Ren Y (2018). Is local platelet-rich plasma injection clinically superior to hyaluronic acid for treatment of knee osteoarthritis? A systematic review of randomized controlled trials. Arthritis Res Ther.

[28] Montañez-Heredia E, Irízar S, Huertas PJ (2016). Intra-articular injections of platelet-rich plasma versus hyaluronic ACID in the treatment of osteoarthritic knee pain: a randomized clinical trial in the context of the Spanish National Health Care System. Int J Mol Sci.

[29] Bennell KL, Paterson KL, Metcalf BR (2021). Effect of intra-articular platelet-rich plasma versus placebo injection on pain and medial tibial cartilage volume in patients with knee osteoarthritis: the RESTORE randomized clinical trial. JAMA.

[30] Ghorbani O, Mahdibarzi D, Yousefi-Tooddeshki P (2024). Comparison of the short-term effect of intra-articular hyaluronic acid and platelet-rich plasma injections in knee osteoarthritis: a randomized clinical trial. J Prev Med Hyg.

[31] Sun H, Zhai H, Han K (2024). Clinical outcomes of autologous adipose-derived mesenchymal stem cell combined with high tibial osteotomy for knee osteoarthritis are correlated with stem cell stemness and senescence. J Transl Med.

[32] Pabinger C, Lothaller H, Kobinia GS (2024). Intra-articular injection of bone marrow aspirate concentrate (mesenchymal stem cells) in KL grade III and IV knee osteoarthritis: 4 year results of 37 knees. Sci Rep.

[33] Baria M, Pedroza A, Kaeding C (2022). Platelet-rich plasma versus microfragmented adipose tissue for knee osteoarthritis: a randomized controlled trial. Orthop J Sports Med.

[34] Gobbi A, Dallo I, D'Ambrosi R (2023). Autologous microfragmented adipose tissue and leukocyte-poor platelet-rich plasma combined with hyaluronic acid show comparable clinical outcomes for symptomatic early knee osteoarthritis over a two-year follow-up period: a prospective randomized clinical trial. Eur J Orthop Surg Traumatol.

[35] Delgado-Enciso I, Paz-Garcia J, Valtierra-Alvarez J (2018). A phase I-II controlled randomized trial using a promising novel cell-free formulation for articular cartilage regeneration as treatment of severe osteoarthritis of the knee. Eur J Med Res.

[36] Totlis T, Achlatis V, Emfietzis PK, Marín Fermín T, Pettas T, Sideridis A, Terzidis I (2026). A single intra-articular stromal vascular fraction with platelet-rich plasma injection yields superior clinical outcomes than a hyaluronic acid injection in patients with knee osteoarthritis: a prospective comparative study. Knee Surg Sports Traumatol Arthrosc.

[37] Irrgang JJ, Anderson AF, Boland AL (2001). Development and validation of the international knee documentation committee subjective knee form. Am J Sports Med.

[38] Karaborklu Argut S, Celik D, Ergin ON, Kilicoglu OI (2024). Does the combination of platelet-rich plasma and supervised exercise yield better pain relief and enhanced function in knee osteoarthritis? A randomized controlled trial. Clin Orthop Relat Res.

